# It’s the network, stupid: a population’s sexual network connectivity determines its STI prevalence

**DOI:** 10.12688/f1000research.17148.2

**Published:** 2019-01-30

**Authors:** Chris R. Kenyon, Wim Delva

**Affiliations:** 1 Institute of Tropical Medicine, Antwerp, Antwerp, 2000, Belgium; 2 Department of Medicine, University of Cape Town, Cape Town, South Africa; 3 Department of Global Health, University of Stellenbosch, Stellenbosch, South Africa; 4 International Centre for Reproductive Health, Ghent University, Ghent, Belgium; 5 South African Centre for Epidemiological Modelling and Analysis, University of Stellenbosch, Stellenbosch, South Africa

**Keywords:** HIV, STI, bacterial vaginosis, sexual network connectivity, concurrency

## Abstract

There is little consensus as to why sexually transmitted infections (STIs), including HIV and bacterial vaginosis (BV) are more prevalent in some populations than others. Using a broad definition of sexual network connectivity that includes both *structural* and *conductivity-related* factors, we argue that the available evidence suggests that high prevalence of traditional STIs, HIV and BV can be parsimoniously explained by these populations having more connected sexual networks. Positive feedback, whereby BV and various STIs enhance the spread of other STIs, then further accentuates the spread of BV, HIV and other STIs. We review evidence that support this hypothesis and end by suggesting study designs that could further evaluate the hypothesis, as well as implications of this hypothesis for the prevention and management of STIs.

## Introduction

**Figure 1.**
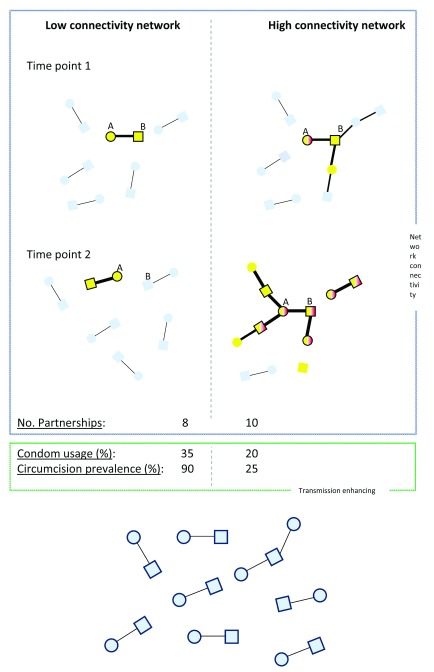
Schematic comparison of STI spread in a low (left) and high (right) sexual network connectivity populations soon after sexual debut. In both populations, STI acquisition commences when ‘A’ has sex with an older man and acquires BV-associated bacteria (yellow) and HSV-2 (black border around each node). In the high connectivity population ‘A’ also acquires *T. vaginalis* (TV; red) from this relationship. The major determinant of the difference in network connectivity is that more relationships run concurrently in the high connectivity population. This facilitates STI spread by: i) Creating a larger reachable path for STIs ^13^, ii) Removing the benefits of partner sequencing seen in serial monogamy (for details see 21), iii) Reducing the time between STI transmissions since infections are not trapped in dyads ^21^ and iv) Bypassing the rapid-clearance-in-males-buffer ^31, 32^. This is the buffer that reduces STI spread in serial monogamous networks where the gap between partnerships (time points 1 and 2) is longer than the duration of colonization of TV and BV-associated bacteria in men. This gap protects the women at time point 2 in the serial monogamy/low connectivity population (represented by the partner of B at time point 2) but not in the high connectivity population from BV and TV acquisition. Various STIs, including HSV-2, BV and TV enhance the susceptibility/infectiousness of other STIs, leading to positive feedback loops. This is conveyed via the transmission probabilities being depicted as proportional to edge width. The high connectivity network also has a low prevalence of circumcision and condom usage which further increase STI transmission probabilities in this population. The combination of high network connectivity, low circumcision/condom-use lead to a rapid spread of multiple STIs in the high- but not the low-connectivity network (blue nodes, no STIs; squares, men; circles, women).

There is little consensus as to why the prevalence of bacterial vaginosis (BV), HIV and other sexually transmitted infections (STIs) varies so dramatically around the world. A range of explanations have been put forward, including variation in circumcision prevalence ^1^, STI treatment efficacy ^2^, poverty ^2– 4^, socioeconomic inequality ^5^, gender inequality ^6^, migration intensity ^7^, hormonal contraception ^8^, vaginal microbiome ^9^, host genetic susceptibility ^10^ and sexual behavior ^11, 12^. We do not dispute that each of these can play a role in differential STI spread. Rather we argue that differential connectivity of sexual networks emerges as a parsimonious dominant explanation for the global variation in STI prevalence, taking a central position in the causal pathway that links all of the above-mentioned risk factors for STI infection ( Figure 1).

## Outline and origins of the network connectivity theory

STIs are transmitted along sexual networks and, as a result, the structural characteristics of these networks determine the speed and extent of STI spread ^13– 15^. These structural characteristics include summary measures of the number of partners per unit time, coital frequency, prevalence of concurrent partnering (having two or more partners at the same time), size of core groups (and their connections with non-core populations), type of sex, size of sexual network, length of gaps between partnerships and degree/type of homophily ^13, 15– 20^ (reviewed in 21). These structural factors determine the forward reachable path of a network, which is defined as the cumulative set of individuals in a population that can be infected with an STI from an initial seed via a path of temporally ordered partnerships ^22^. Two particularly important determinants of the forward reachable path are the prevalence of concurrency and the number of partners per unit time ^22^.

**Figure 2.**
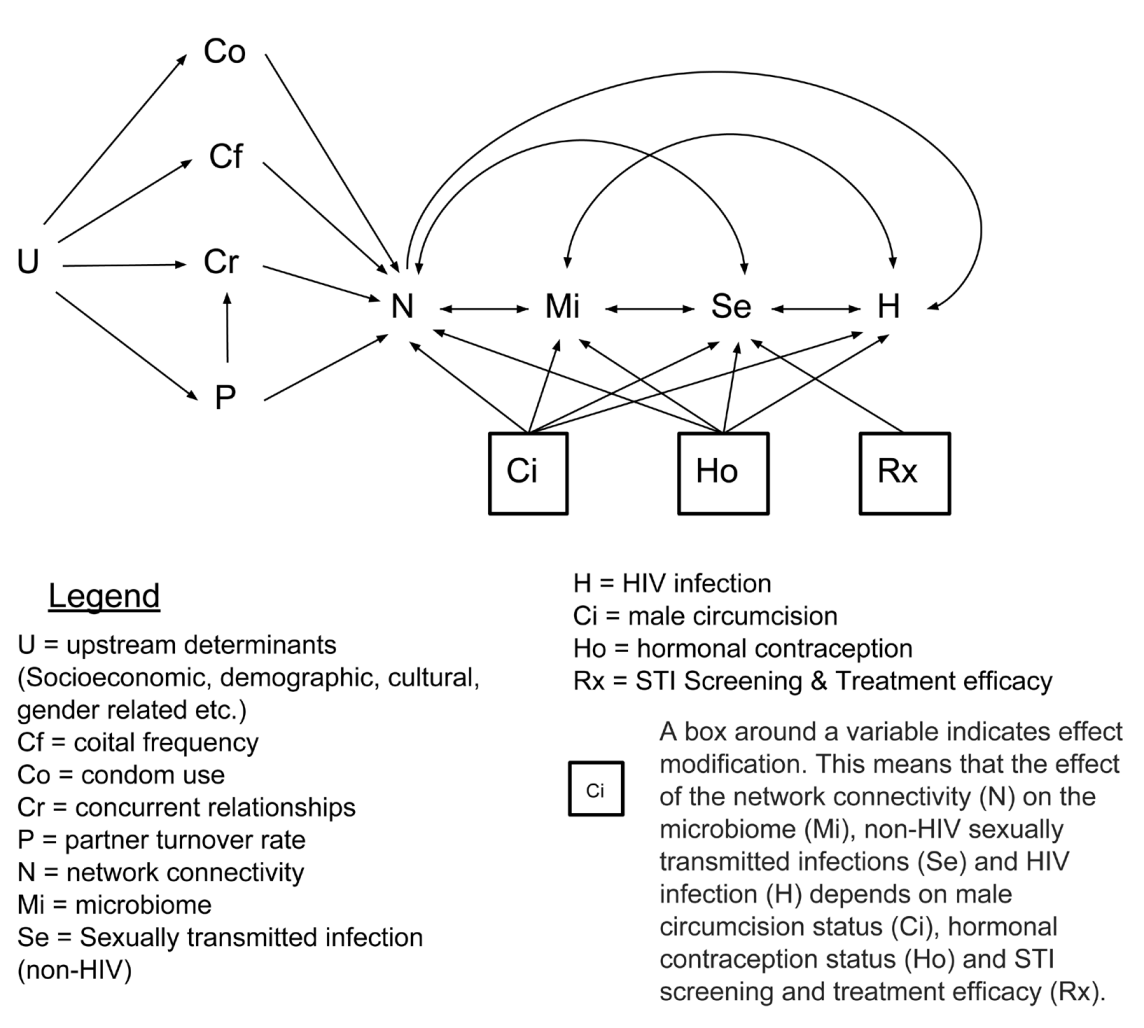
Conceptual framework for the understanding the genesis of differences in STI prevalence between populations. Note that we use directed cyclic graphs as short-hand notation for an infinite acyclic directed graph containing variables indexed by time. Our definition of network connectivity is broad: in addition to considering the sexual links between individuals, it takes into account the “conductivity” and timing of these links.

STI transmission can also be enhanced through a sexual network by factors that enhance the conductivity or probability of STI transmission per sex act. These factors include a low prevalence of circumcision, pre-exposure prophylaxis (PrEP) and condom usage ( Figure 1 and Figure 2). Enhanced screening/early and effective treatment of STIs could reduce spread of STIs via reducing the duration of infectivity. Because numerous STIs enhance the transmission/acquisition of other STIs ^23^, effective STI control could then also reduce the conductivity of a network. We use a broad definition of network connectivity in this paper that includes both these *structural* and *conductivity-related* factors.

The origins of this network connectivity theory lie in the STI modeling field. Previous modelling studies from the 1970s established the importance of the rate of partner change and mixing between core and non-core groups to STI spread ^24, 25^. Seminal modelling papers by Morris *et al.* ^13^ and Watts *et al.* ^15^ in the 1990s built on these findings by revealing that the prevalence of sexual partner concurrency may be a particularly important determinant of network connectivity. Their analyses found that relatively small increases in concurrency could lead to dramatic increases in network connectivity and as a result, HIV spread ^13^. The main mechanisms whereby concurrency promotes STI spread are illustrated in Figure 1. A number of empirical studies have subsequently established that markers of network connectivity such as concurrency and rate of partner change are correlated with the prevalence of all major STIs ( Table 1). In this paper, we review some of the cross sectional and longitudinal evidence that two components of network connectivity (concurrency and rate of partner change) are associated with STI prevalence. We then summarize evidence that network connectivity influences the prevalence of BV and end by noting the potential for positive feedback loops between various STIs being underpinned by network connectivity.

**Table 1.** A non-exhaustive tabulation of studies that have found number of partners and partner concurrency to be positively associated with various sexually transmitted infections (STIs). Studies that found no association are not included.

STI	No. sex partners		Concurrency prevalence	
	Individual level	Population level	Individual level	Population level
BV	[ 33]	NA	[ 31]	[ 34]
Chlamydia	[ 35, 36]	[ 35]	[ 18, 36, 37]	NA
Gonorrhoea	[ 38]	NA	[ 39]	NA
HIV	[ 40, 41]	[ 42– 44]	[ 45– 47]	[ 48– 50]
HSV-2	[ 51, 52]	NA	[ 52]	[ 53]
HPV	[ 54, 55]	NA	[ 56]	NA
Syphilis	[ 35]	NA	[ 57]	[ 58]
Trichomoniasis	[ 37, 59]	NA	[ 32, 37]	[ 60]

No. of sex partners refers to number of partners over lifetime or over past year. Concurrency refers to the prevalence of concurrency (male, female or combined) for the population level studies and partner concurrency in the individual level studies. NA, not available/no studies found that evaluated this association.

## Markers of network connectivity are correlated with the prevalence of STIs: cross-sectional evidence

*a. Ethnic group comparative analyses*

**Figure 3.**
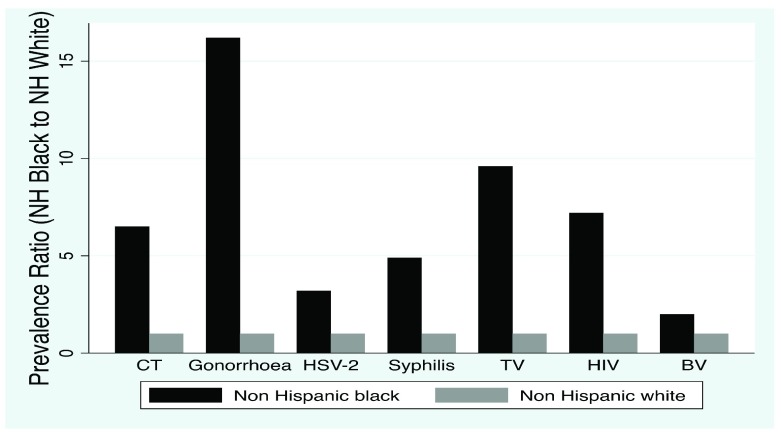
Estimated prevalence ratio of HIV and other sexually transmitted infections in non-Hispanic Blacks versus non-Hispanic Whites in the United States National Health and Nutrition Examination Survey 2003–2004. Figure modified from 12 and 61.

USA: In the United States the prevalence of BV, HIV and most STIs for non-Hispanic blacks in the 1990s was considerably higher than in non-Hispanic whites ( Figure 3). Historical data is limited but the available data demonstrates that these divergences in prevalence extend back to the 1930s for syphilis ^26, 27^ and the 1970s for HSV-2 ^28^. Morris *et al.* used five large national behavioural surveys to investigate which possible risk factors could underpin these differences in HIV prevalence, and found that the prevalence of concurrency was on average 3.5 and 2.1 times higher in non-Hispanic black men and women, respectively. In their modelling analysis, they found that these differences in concurrency prevalence between these groups translated into 2.6-fold differences in HIV prevalence. They did not, however, model the enhanced transmission probability that is associated with acute HIV which subsequent analyses have shown to have a synergistic effect with concurrency on HIV transmission ^29^. Subsequent studies have demonstrated that concurrency plays an important role in the spread of the other STIs and thus the differential concurrency prevalence they found could represent a parsimonious explanation for the differences in the range of STI prevalence demonstrated in Figure 3.

Southern Africa: The HIV prevalence varies 40-fold between ethnic groups in South Africa ^30^. Analyses from 5 nationally representative behavioural surveys revealed that the most plausible risk factors that could explain this were the 5- to 17-fold higher prevalence of male concurrency and the higher number of partners per year in the highest compared to the lowest HIV prevalence ethnic group ^30^. A modelling study likewise demonstrated that the combination of concurrency and rate of partner change was responsible for approximately 75% of the HIV infections in the 1990s when antenatal HIV prevalence increased from 0.7% to 24.5% ^62^. In a similar vein, modelling studies from Zimbabwe found that both the observed high prevalence of concurrency and the increased transmission probability associated with acute HIV were needed to replicate Zimbabwe's explosive HIV epidemic curve ^29^.

Elsewhere: The prevalence of concurrency and/or number of partners have also been found to be associated with variations in HIV prevalence by ethnic group in Ethiopia ^43^, Honduras ^63^, Kenya ^64^, Uganda ^65^ and the United Kingdom ^66– 68^. Although no published study has assessed how generalizable these findings are globally, one study attempted to do this within sub-Saharan African countries. This study used demographic and health surveys to systematically assess the behavioural correlates of HIV prevalence by region (as a proxy for ethnic group) in 47 surveys from 27 African countries where HIV prevalence varied by at least two-fold between regions. It found that the lifetime number of partners reported by men and women was positively correlated with HIV prevalence in 23 and 18 out of 36 surveys, respectively. Likewise, reporting sex with a non-marital, non-cohabiting partner by men and women was positively correlated with HIV prevalence in 38 and 39 out of 47 surveys, respectively ^69^.

### The relationship between ethnicity, race and STI prevalence

It is of paramount importance to emphasize that our hypothesis makes no reference to race. The hypothesis proposes that there are differences in sexual behavior between different groups of people which translate into differences in network connectivity and as a result differential STI prevalence. These groups can be defined by sexual orientation, ethnicity, social class, caste or whatever categories meaningfully segregate sexual networks. These categories are social constructs and thus vary considerably across time and place. It is our considered opinion that investigators who conduct investigations into STI epidemiology usingthese categories do so with sufficient sensitivity to the concerns as to how these categories are and have been used and abused.

Other authors have hypothesized that biological differences between racial groups play an important role in STI epidemiology. A recent form of this argument is that ‘black populations’ are innately more likely to have bacterial-vaginosis-type vaginal microbiomes which in turn facilitates the transmission of various STIs in this population ^9^. We and others have argued that the evidence does not support this, and other race-based explanations of differential STI spread ^70, 71^. As an example, we noted that ‘black populations’ with evidence of low sexual network connectivity have a very low prevalence of BV and conversely ‘white populations’ with high connectivity had a high BV prevalence ^70, 71^.

*b. Country level comparisons*

In the country level analysis we focus on studies that investigate the correlates of country level peak HIV prevalence. Peak HIV prevalence, which represents the maximal HIV prevalence that countries obtained prior to the widespread availability of antiretroviral therapy is a useful composite measure of the factors that enabled the rapid spread of HIV ^72^. Peak HIV prevalence is based on generally high quality data on HIV prevalence around the world. National HIV prevalence estimates are frequently based on nationally representative serosurveys and other sources such as antenatal surveys that involve sample sizes of up to 216,703 individuals ^73– 75^. Nationally representative, HIV serolinked, demographic health surveys are one example of this. MeasureDHS provides open-access to 72 such surveys from 37 countries ( https://dhsprogramme.com). As such, peak HIV prevalence offers a useful outcome measure to assess what the correlates of rapid and extensive HIV transmission are ^72^. Studies have shown that peak HIV prevalence is not associated with a number of risk factors widely believed to be important for HIV spread: poverty, socioeconomic inequality, gender inequality, prevalence of migration and STI treatment efficacy ^6, 11, 76, 77^. These findings are of considerable consequence. Many authors have claimed that STIs are diseases of poverty ^2– 4^. Studies from Africa and elsewhere suggest that this is far from universally the case. Using HIV-serolinked and nationally representative survey data from eight countries in sub-Saharan Africa, Mishra *et al.*, for example, established that HIV prevalence increased monotonically with wealth quintile for both men and women ^78^. This finding has since been confirmed in 19 other countries ^40^.

Only two risk factors have been consistently found to be associated with peak HIV prevalence: circumcision and the prevalence of concurrency.

i. *Circumcision:* There is a strong negative association between circumcision and peak HIV prevalence within sub-Saharan Africa, but not globally ^11, 77^. This is unsurprising since sub-Saharan Africa has the highest prevalence of HIV and the second highest prevalence of circumcision in the world ^77, 79^. The vast majority of the world’s population lives in countries with both low HIV and low circumcision prevalence ^11^. Various lines of evidence suggest that something else is driving the spread of HIV in sub-Saharan Africa and that circumcision is then moderating this risk ^77, 80^.

ii. *Concurrency prevalence*: The prevalence of male concurrency has been found to be associated with peak HIV prevalence in a cross country study ^48^. Other studies have however failed to find this association ^81, 82^ but serious methodological questions have been raised pertaining to these studies including the fact one of these studies compared 5 year cumulative concurrency rates from European countries with point prevalence of concurrency in African countries ^48, 81, 83^. A further problem related to these cross-national comparisons is that national populations are frequently composed of multiple subpopulations that may have large differences in HIV prevalence. In 29 sub-Saharan countries with available data, for example, HIV prevalence was found to vary by a median of 3.7-fold (IQR 2.9-7.9) between regions within countries ^69^. As argued above, more fine-grained studies investigating the correlates of HIV prevalence by ethnic group or region within these and other countries have found a range of markers of network connectivity (such as partner number and concurrency) and other risk factors to be associated with HIV prevalence ^12, 43, 65, 66^.

*c. Men who have sex with men (MSM) vs. heterosexual comparison*

A number of high-income countries are experiencing epidemics of a range of STIs that are disproportionately affecting MSM ^84^. In the year 2014 in London, for example, MSM who comprised only 2% of the population, contributed a disproportionate number of diagnoses of STIs (23%/63%/69%/90% of all new chlamydia/HIV/gonorrhoea/syphilis, respectively) ^85^. Lymphogranuloma venereum (LGV) and sexually transmitted hepatitis C have also been noted to disproportionately affect MSM in contemporary outbreaks in high-income countries ^84, 86^. A parsimonious explanation for this clustering of STIs in MSM is a combination of behavioural factors including number of sex partners and partner concurrency ^86, 87^. In the United Kingdom, MSM in 2012 reported a median (interquartile range) of 5 (2–30) partners in the past 5 years versus 1 (1–3) reported by heterosexual men ^88^; likewise, the proportions reporting concurrency in the prior 5 years were 52% and 15% for these two groups, respectively ^88^.

## Markers of network connectivity are correlated with the incidence of STIs: longitudinal evidence

In this section, we consider two (of many possible) examples where large changes in STI incidence are preceded by corresponding changes in network connectivity.

*a) Incidence of primary/secondary syphilis in MSM in the USA 1963 to 2013.*

**Figure 4.**
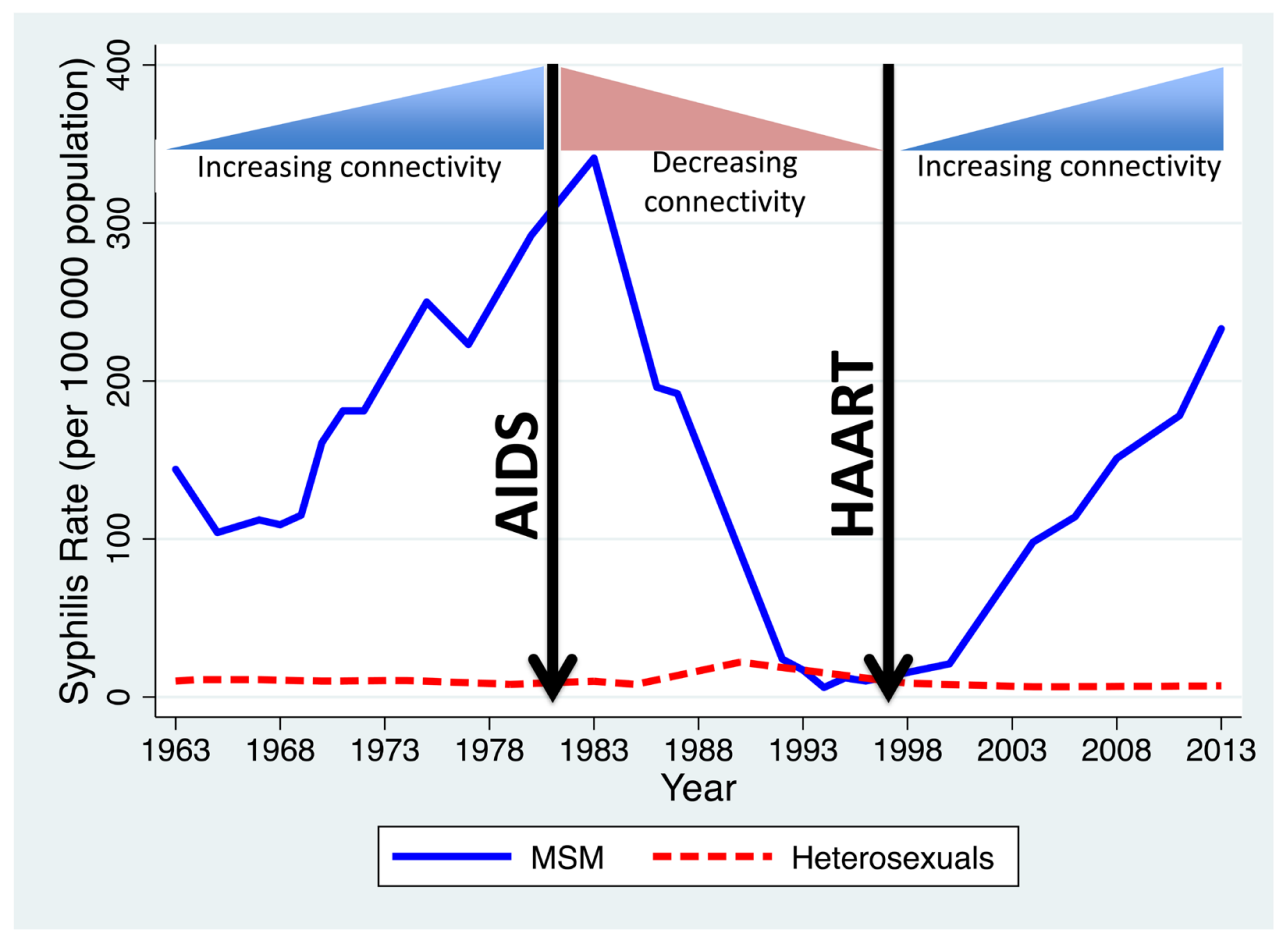
Fluctuations in estimated annual rate of primary/secondary syphilis among men who have sex with men (MSM) and men who have sex with women only (MSW) in the USA, 1963–2013 (Modified from 89). Also shown is how these were temporally associated with changes in network connectivity represented schematically.

In their review of syphilis epidemiology in the United States 1963 to 2013, Peterman *et al.*, found evidence of an initial dramatic increase in primary/secondary syphilis in MSM between 1963 and 1982 followed by a steep decline to close to zero cases in 1994 and a subsequent increase to 228/100,000 in 2013 ( Figure 4) ^89^. Increases in multiple partnering were thought to underpin the initial increase ^89– 92^. The AIDS epidemic in the 1980s led to reductions in network connectivity via both behaviour change and deaths of individuals (from AIDS) centrally placed in sexual networks ^89, 93^. The arrival of effective antiretroviral therapy from 1996 onwards played an important role in the increases in rates of partner change and reductions in condom usage ^89, 94^ noted during the ongoing epidemic of syphilis in this population. Although this evidence is indirect and susceptible to confounding, it is at least suggestive that the two large increases and one precipitous decline in syphilis incidence were determined to some extent by corresponding changes in network connectivity. Of note, this epidemic trajectory of syphilis in MSM in the United States was similar to that of a range of other STIs such as LGV and gonorrhoea in this same population and in MSM in other high income countries ^84^. In a range of European countries where MSM were similarly affected by the AIDS epidemic, the incidence of STIs such as syphilis, LGV and gonorrhoea declined to very low rates in the post AIDS period before large increases in the late 1990s onwards corresponding to increases in partner number and declines in condom usage ^84^.

*b) Southern and Eastern Africa*

A number of studies from general populations in Southern and Eastern Africa have concluded that reductions in partner number and concurrency played an important role in the impressive declines in HIV incidence in Uganda, Zimbabwe and other countries in the region ^95– 100^. Delayed sexual debut, increased condom usage, enhanced antiretroviral therapy coverage and AIDS mortality (via reduced network connectivity) also played an important role in this regard ^96, 100, 101^.

## Clustering of STIs includes incurable STIs and network connectivity is the most parsimonious way to explain this clustering

We have already noted the striking clustering of STIs within certain ethnic groups and sexual orientations in a number of countries. Strong evidence of clustering of STIs has also been found at WHO world regional ^102^ and country levels. At a country level, the peak HIV prevalence has been found to be associated with the prevalence of a range of STIs before/early in the HIV epidemics: syphilis ^103^, gonorrhoea ^104^, HSV-2 ^103^ and trichomoniasis ^104^ and BV ^104^.

This clustering of STIs is important for two reasons. Firstly, it suggests that one or more common risk factors could underpin variations in all these STIs. Secondly, the treatable but incurable STI, HSV-2 is correlated with both peak HIV prevalence ^103^ and antenatal syphilis prevalence from the pre-HIV period ^66^. This is relevant because differential STI treatment efficacy can explain differences in the prevalence of treatable STIs such as syphilis but not HSV-2. Differential network connectivity, which can explain the differential spread of all STIs, is thus a more parsimonious way to explain the clustering of STIs.

## Network connectivity is also a risk factor for BV

Recent couple studies have demonstrated that the consortia of bacteria that constitute the dysbiosis, BV are sexually transmitted ^105– 111^. BV is strongly associated with number of sexual partners and reporting partner concurrency at an individual level ^31, 33, 112, 113^. An ecological study found associations at the level of countries between the prevalence of male concurrency and the prevalence of BV ^34^. The same association was also found at the level of ethnic groups within countries ^34^.

Populations with high network connectivity are thus likely to have a high prevalence of BV which in addition to the adverse clinical effects of BV ^114^, is important because of the positive feedback cycles between BV and the traditional STIs. BV for example has been shown to enhance susceptibility to chlamydia ^115, 116^, gonorrhoea ^115^, HIV ^117, 118^, HSV-2 ^119, 120^ and TV ^115, 116, 121^. HSV-2 and TV have in turn been shown to increase the risk for acquisition of BV and other STIs ^115– 117, 122^. A more detailed review of the evidence linking BV prevalence to network connectivity is provided in 71.

Previous modelling studies have found that relatively small increases in parameters of network connectivity can lead to non-linear increases in HIV/STI spread ^13^. If this applies to BV as well, then more connected sexual networks would be expected to facilitate the rapid spread of BV and the various STIs soon after sexual debut. These would then increase susceptibility and transmission of other STIs, adding a further means by which enhanced network connectivity could lead to increases in STI spread. Network connectivity would thus indirectly enhance probability of transmission per sex act for different STIs ( Figure 1).

## Limitations

It should be emphasized that this paper presents a narrative, non-systematic review of evidence for network connectivity as a parsimonious explanation of variations in genital microbiomes and STI prevalence. As such, our sampling of evidence is likely biased. We acknowledge that we have picked evidence that is supportive of our hypothesis. Our definition of network connectivity could also be criticized as being impractical because it includes such a breadth of structural and conductivity variables. Consequently, in our conceptual framework of network connectivity, different combinations of these variables could yield the same STI prevalence.

Considerable further work is necessary to construct formulae of the determinants of network connectivity and then establish how these relate to empirical estimates of STI prevalence around the world. A global study that uses a standardized methodology ( Table 2) to map the variations in STI prevalence and associated risk factors by ethnic group/region within all relevant countries could provide valuable further information. So too, longitudinal studies that follow up populations from high and low STI prevalence populations from the time of sexual debut would be useful. These should accurately map the timing and correlates of STI spread including alterations of vaginal and penile microbiomes and allow more precise quantitation of which risk factors are most important for STI spread. These studies should enable the construction of more accurate models of STI spread that can be used to predict STI prevalence for specific populations under various counterfactual scenarios such as reductions in the prevalence of concurrent partnering.

**Table 2.** Suggested standardized methodology for mapping variations in sexually transmitted infection (STI) prevalence and associated risk factors by ethnic group/region in all relevant countries.

No.	Methodology
1	Relevant countries are those where STI/HIV prevalence varies by 2-fold between ethnic groups/regions
2	Assess if analysis is more appropriately broken down by ethnic group or region based on: 1. HIV prevalence differential greater by region or by ethnicity 2. Degree of homophily by ethnicity in partner choice
3	Use hierarchy of survey designs for data analysis, with HIV and other STI serolinked, nationally, regionally and ethnic group representative sampling as the gold standard
4	Use standardized definitions of variables such all 15–49 year olds and all 15–24 year olds regardless of if ever had sex or not
5	Standardized methodology for data analysis and visualization

Our theory includes mention of the wide array of upstream socioeconomic and political factors that have been shown to influence the spread of STIs ^123^. We argue that the pathways through which these factors facilitate STI transmission is to a large extent mediated via alterations in network connectivity ^124^. We have not, however, gone into any detail into reviewing the evidence on which this view is based ^123, 125, 126^. Furthermore, our focus on the more downstream factors responsible for STI transmission should not detract from efforts to target the upstream determinants of enhanced STI transmission.

## Implications of network connectivity: Know Your Network, Determine Your Prevalence

If confirmed by further experimental data, the network connectivity approach would generate new opportunities for STI prevention interventions. Whilst individual level biomedical STI control interventions have delivered considerable successes, they do not address the root cause of high STI prevalence and are therefore unlikely to accomplish radical prevention ^126, 127^. HIV pre-exposure prophylaxis and treatment as prevention, for example, may reduce HIV transmission but will not reduce the transmission of other STIs. If differential network connectivity is a fundamental determinant of STI and BV prevalence then this could be communicated to affected populations as an opportunity to effect radical prevention. Along these lines, a ‘Know your Network’ intervention has been successfully piloted in Kenya ^128^. During a community meeting, the community's sexual network was computed by fitting a dynamic network model to data from individual sexual diaries, and a graphical representation of the network was fed back to the community. Participants reported the intervention to be transformative but formal trials are required to assess the efficacy on STI incidence of this type of intervention ^128^. Uganda’s ‘Zero Grazing’ campaign ^129^ and similar processes elsewhere in Africa ^100, 130^ which resulted in dramatic declines in side-partners and HIV incidence, could be viewed as providing both guidance and evidence for this approach.

## Data availability

No data are associated with this article.
