# Supplementary material for: It’s the network, stupid: a population’s sexual network connectivity determines its STI prevalence
Source: F1000Res. 2019 Jan 30;7:1880. Originally published 2018 Dec 2. [Version 2] doi: 10.12688/f1000research.17148.2 (PMC6376253; doi:10.12688/f1000research.17148.2)
Supplement: Peer review history

# Peer review: Referee response for version 2

**DOI:** 10.5256/f1000research.19714.r43791

**Published:** 2019-02-13

**Authors:** Ann M. Jolly

**Affiliations:** 1 School of Epidemiology, Public Health and Preventative Medicine, University of Ottawa, Ottawa, ON, Canada

Authors have kindly resolved all my concerns so it is fully approved by me.

I have read this submission. I believe that I have an appropriate level of expertise to confirm that it is of an acceptable scientific standard.

# Peer review: Referee response for version 1

**DOI:** 10.5256/f1000research.18748.r42433

**Published:** 2019-01-11

**Authors:** Eline L Korenromp

**Affiliations:** 1 Avenir Health, Geneva, Switzerland

I agree with the earlier comments by Ann Jolly, and am adding only a few minor additional comments here, meant for clarification.

 Legend of Figure 2: Please clarify or rephrase 'if one conditions on this variable.'

 Page 5, section(b), left column: 'generally high-quality longitudinal data on HIV prevalence around the world': I think these prevalence surveys are not really longitudinal (which commonly denotes cohort data, where a cohort of identified individuals is followed for incidence at individual-level), but rather periodic (in a similar population, but with some turnover of individuals). Please rephrase here - and in a similar instance later on in the article.

I have read this submission. I believe that I have an appropriate level of expertise to confirm that it is of an acceptable scientific standard.

# Peer review: Referee response for version 1

**DOI:** 10.5256/f1000research.18748.r41827

**Published:** 2019-01-03

**Authors:** Ann M. Jolly

**Affiliations:** 1 School of Epidemiology, Public Health and Preventative Medicine, University of Ottawa, Ottawa, ON, Canada

This is a well referenced, very interesting paper which attempts and in large part succeeds, in critically examining, and in many cases rejecting common “risk factors/ determinants” of high STI (sexually transmitted infection) rates. The authors have traced a long history in mathematical modelling of primarily HIV and summarised the most common and consistently found explanations of “peak HIV” rates at an ecological level.

 I think this article is thought provoking and even if one is not entirely convinced by it, it adds much deeper and more critical thought to assumptions of mathematical modellers and epidemiologists about the immediate causes of HIV than I have ever seen. I am glad it is published.

 I think the paper could be improved in general by addressing two major areas.  First, some mention of the empirical study of sexual networks should be made.There are examples from all over the world which the authors may use to strengthen their ultimate hypothesis; that sexual interactions between sexual network members in which some people are infected with STI are the primary and necessary conditions for STI to transmit ^1^^,^^2^. This will help define for their readers very clearly the fact that because ecological findings focus on whole systems, whether national or regional, these studies are more indicative of large networks and should not be regarded as “weaker” evidence as they are in epidemiology.  The authors can also argue that the ultimate risk factor on an individual level for acquiring gonorrhoea for example is having unprotected sex with another person with gonorrhea; that is it and that is all. Other factors are moderators of that plain risk; such as use of condoms, cumulative frequency of intercourse, etc. Concurrency or number of sex partners are indirect indicators of the risk itself; because transmission of STI is an interaction which occurs between at minimum two people, the other “factors” are reduced to characteristics or behaviors of individuals within the population, which again are proxies, though more proximal than age, sex, ethnic background or income.

 The second thing I would be very clear on is the part played by ethnic group. This is again just a proxy for sexual interactions and cultural norms, and may not be a consistent nor accurate marker.  However, it is very easy in the discussion of HIV in Africa to “racialize” the epidemic and/or behavior.  For example, the effect of the labour policies under apartheid South Africa, where black labourers were forced into a pattern of migrant labor for a year at a time from their rural homes to large urban or mining compounds did much to accelerate sexual interactions which in turn exacerbated HIV spread. Likewise, sexual violence in some parts of Africa is extremely high, and while it is not mentioned here specifically, certainly does contribute to transmission. However, this also is linked to harsh colonial conditions and the authors could make it very clear that this should not be interpreted as being a intrinsically ethnic or cultural characteristic. The successful Ugandan educational intervention is a great demonstration of that. One of the best network explanations of different STI rates in people of different ethnic backgrounds can be found in ^3^, pages 689-697.

 More minor comments follow;

 Page 2 - I love Figure 1.

 Page 3, 2nd paragraph - better give credit where credit is due ^4^.

 Page 3, 3rd ^^paragraph - so concurrency may be a marker of connectivity; or a determinant, but one may equally pose that if one has many sex partners the only way to fit them all in is to have concurrent ones.. and that may have an effect also on frequency of intercourse with each partner. Lots of food for thought!

 Page 4 - wonderful summary! I would love to see a systematic review which includes an exhaustive list with negative findings. Again, one could look for only proximal causes of transmission rather than secondary or structural determinants.

 Page 5 - you state that “Peak high prevalence HIV is based generally of high quality longitudinal data”. Please justify and provide references.

 Page 5 - the statements on poverty and socioeconomic status, gender inequality which are not consistently associated with HIV are great; this is because these determinants are only distally linked to transmission.  

 Page 5, paragraph 8 - to describe numbers of partners more accurately I would use the median, range or IQR as the mean implies these distributions are Gaussian, but they are not. The devil is literally in "de tail"  Also one may have to look at the penile anal penetration risk of transmission which is not exactly comparable to penile vaginal sex. But I agree in principle.

 Page 7, paragraph 1 - I would be more accurate here, HSV is certainly treatable, but not as easily diagnosed or treated as the bacterial STI. 

 Page 7, Table 2 - you may find the discussion of ethnic homophily and heterogeneity in networks interesting in ^1^^,^^2^.  

 Great paper, good thinking, and great to challenge convention!

I have read this submission. I believe that I have an appropriate level of expertise to confirm that it is of an acceptable scientific standard, however I have significant reservations, as outlined above.

## References

[1] JollyAMMuthSQWylieJLPotteratJJ: Sexual networks and sexually transmitted infections: a tale of two cities. *J Urban Health*.2001;78(3) : 10.1093/jurban/78.3.433 433-45 10.1093/jurban/78.3.433 11564847PMC3455915

[2] The New Public Health and STD/HIV Prevention.2013; 10.1007/978-1-4614-4526-5 10.1007/978-1-4614-4526-5

[3] MokotoffE: African Americans and HIV/AIDS: Understanding and Addressing the Epidemic. *JAMA*.2011;305(18) : 10.1001/jama.2011.609 10.1001/jama.2011.609

[4] WasserheitJN: Epidemiological synergy. Interrelationships between human immunodeficiency virus infection and other sexually transmitted diseases. *Sex Transm Dis*.19(2) :61-77 1595015

# Response

**Published:** 2019-01-22

**Authors:** Chris Kenyon

**Affiliations:** Institute of Tropical Medicine, Antwerp, Belgium

This is a well referenced, very interesting paper which attempts and in large part succeeds, in critically examining, and in many cases rejecting common “risk factors/ determinants” of high STI (sexually transmitted infection) rates. The authors have traced a long history in mathematical modelling of primarily HIV and summarised the most common and consistently found explanations of “peak HIV” rates at an ecological level.

I think this article is thought provoking and even if one is not entirely convinced by it, it adds much deeper and more critical thought to assumptions of mathematical modellers and epidemiologists about the immediate causes of HIV than I have ever seen. I am glad it is published.

I think the paper could be improved in general by addressing two major areas.  First, some mention of the empirical study of sexual networks should be made.There are examples from all over the world which the authors may use to strengthen their ultimate hypothesis; that sexual interactions between sexual network members in which some people are infected with STI are the primary and necessary conditions for STI to transmit ^1^ ^,^ ^2^. This will help define for their readers very clearly the fact that because ecological findings focus on whole systems, whether national or regional, these studies are more indicative of large networks and should not be regarded as “weaker” evidence as they are in epidemiology.  The authors can also argue that the ultimate risk factor on an individual level for acquiring gonorrhoea for example is having unprotected sex with another person with gonorrhea; that is it and that is all. Other factors are moderators of that plain risk; such as use of condoms, cumulative frequency of intercourse, etc. Concurrency or number of sex partners are indirect indicators of the risk itself; because transmission of STI is an interaction which occurs between at minimum two people, the other “factors” are reduced to characteristics or behaviors of individuals within the population, which again are proxies, though more proximal than age, sex, ethnic background or income.

The second thing I would be very clear on is the part played by ethnic group. This is again just a proxy for sexual interactions and cultural norms, and may not be a consistent nor accurate marker.  However, it is very easy in the discussion of HIV in Africa to “racialize” the epidemic and/or behavior.  For example, the effect of the labour policies under apartheid South Africa, where black labourers were forced into a pattern of migrant labor for a year at a time from their rural homes to large urban or mining compounds did much to accelerate sexual interactions which in turn exacerbated HIV spread. Likewise, sexual violence in some parts of Africa is extremely high, and while it is not mentioned here specifically, certainly does contribute to transmission. However, this also is linked to harsh colonial conditions and the authors could make it very clear that this should not be interpreted as being a intrinsically ethnic or cultural characteristic. The successful Ugandan educational intervention is a great demonstration of that. One of the best network explanations of different STI rates in people of different ethnic backgrounds can be found in ^3^, pages 689-697.

**Reply:**

**In addition to small additions to the text along the lines suggested we have also added the two references suggested and the following new paragraphs:**

Page 6, L28:

**The relationship between ethnicity, race and STI prevalence**

It is of paramount importance to emphasize that our hypothesis makes no reference to race. The hypothesis proposes that there are differences in sexual behavior between different groups of people which translate into differences in network connectivity and as a result differential STI prevalence. These groups can be defined by sexual orientation, ethnicity, social class, caste or whatever categories meaningfully segregate sexual networks. These categories are social constructs and thus vary considerably across time and place. It is our considered opinion that investigators who conduct investigations into STI epidemiology using these categories do so with sufficient sensitivity to the concerns as to how these categories are and have been used and abused.

Other authors have hypothesized that biological differences between racial groups play an important role in STI epidemiology. A recent form of this argument is that ‘black populations’ are innately more likely to have bacterial-vaginosis-type vaginal microbiomes which in turn facilitates the transmission of various STIs in this population ^1^. We and others have argued that the evidence does not support this, and other race-based explanations of differential STI spread ^2,3^. As an example, we noted that ‘black populations’ with evidence of low sexual network connectivity have a very low prevalence of BV and conversely ‘white populations’ with high connectivity had a high BV prevalence ^2,3^.

Page 10, L22:

Our theory includes mention of the wide array of upstream socioeconomic and political factors that have been shown to influence the spread of STIs ^4^. We argue that the pathways through which these factors facilitate STI transmission is to a large extent mediated via alterations in network connectivity ^5^. We have not, however, gone into any detail into reviewing the evidence on which this view is based ^4,6,7^. Furthermore, our focus on the more downstream factors responsible for STI transmission should not detract from efforts to target the upstream determinants of enhanced STI transmission.

More minor comments follow;

Page 2 - I love Figure 1.

Page 3, 2nd paragraph - better give credit where credit is due ^4^.

**Reply:**

**This reference has been added**

Page 3, 3rd paragraph - so concurrency may be a marker of connectivity; or a determinant, but one may equally pose that if one has many sex partners the only way to fit them all in is to have concurrent ones.. and that may have an effect also on frequency of intercourse with each partner. Lots of food for thought!

**Reply:**

**Indeed increasing concurrency tends to lead to an increase in number of partners per unit time. Interestingly a number of modelling studies have shown that, in certain scenarios, increasing concurrency whilst keeping total number of partnership per unit time unchanged can still result in increases in markers of connectivity such as the forward reachable path** **^8,9^** **. In the datasets we have reviewed, the two however tend to covary at population levels** **^10-13^** **and thus in our estimation a reasonable case can be made to not consider these two variables in isolation.**

Page 4 - wonderful summary! I would love to see a systematic review which includes an exhaustive list with negative findings. Again, one could look for only proximal causes of transmission rather than secondary or structural determinants.

**Reply:**

**Hopefully someone reading this will be tempted to do this review.**

Page 5 - you state that “Peak high prevalence HIV is based generally of high quality longitudinal data”. Please justify and provide references.

**Reply:**

**A justification for this assertion has been provided and backed up with 3 new references (Page 7, L9-14).**

Page 5 - the statements on poverty and socioeconomic status, gender inequality which are not consistently associated with HIV are great; this is because these determinants are only distally linked to transmission.  

**Reply:**

**Thank you.**

Page 5, paragraph 8 - to describe numbers of partners more accurately I would use the median, range or IQR as the mean implies these distributions are Gaussian, but they are not. The devil is literally in "de tail"  Also one may have to look at the penile anal penetration risk of transmission which is not exactly comparable to penile vaginal sex. But I agree in principle.

**Reply:**

**Thank you for pointing this out. We agree and have changed the figures provided to medians and interquartile ranges.**

Page 7, paragraph 1 - I would be more accurate here, HSV is certainly treatable, but not as easily diagnosed or treated as the bacterial STI. 

**Reply:**

**This has been changed as suggested.**

Page 7, Table 2 - you may find the discussion of ethnic homophily and heterogeneity in networks interesting in ^1^ ^,^ ^2^.  

**Reply:**

**Thank you for these references which have been added to the paper.**

References

1.         Buve A, Jespers V, Crucitti T, Fichorova RN. The vaginal microbiota and susceptibility to HIV. *AIDS. *2014;28(16):2333-2344.

2.         Kenyon C, Osbak K. Sexual networks, HIV, race and bacterial vaginosis. *AIDS. *2015;29(5):641-642.

3.         Kenyon CR, Delva W, Brotman RM. Differential sexual network connectivity offers a parsimonious explanation for population-level variations in the prevalence of bacterial vaginosis: a data-driven, model-supported hypothesis. *BMC Womens Health. *2019;19(1):8.

4.         Aral SO. Determinants of STD epidemics: implications for phase appropriate intervention strategies. *Sex Transm Infect. *2002;78 Suppl 1:i3-13.

5.         Gorbach PM, Stoner BP, Aral SO, WL HW, Holmes KK. "It takes a village": understanding concurrent sexual partnerships in Seattle, Washington. *Sex Transm Dis. *2002;29(8):453-462.

6.         Aral SO, Leichliter JS. Non-monogamy: risk factor for STI transmission and acquisition and determinant of STI spread in populations. *Sex Transm Infect. *2010;86 Suppl 3:iii29-36.

7.         Aral SO, Leichliter JS, Blanchard JF. Overview: the role of emergent properties of complex systems in the epidemiology and prevention of sexually transmitted infections including HIV infection. *Sex Transm Infect. *2010;86 Suppl 3:iii1-3.

8.         Morris M, Kretzschmar M. Concurrent partnerships and the spread of HIV. *AIDS. *1997;11(5):641-648.

9.         Morris M, Goodreau S, Moody J. Sexual networks, concurrency and STD/HIV. In: Holmes KK, ed. *Sexually transmitted diseases.* 4th ed. New York: McGraw-Hill Medical; 2008:xxv, 2166 p.

10.        Kenyon C, Buyze J, Colebunders R. HIV prevalence by race co-varies closely with concurrency and number of sex partners in South Africa. *PLoS One. *2013;8(5):e64080.

11.        Kenyon CR. HIV prevalence by ethnic group covaries with prevalence of herpes simplex virus-2 and high-risk sex in Uganda: An ecological study. *PLoS One. *2018;13(4):e0195431.

12.        Kenyon C, Menten J, Vu L, Maughan Brown B. Male circumcision and sexual risk behaviors may contribute to considerable ethnic disparities in HIV prevalence in Kenya: an ecological analysis. *PLoS One. *2014;9(8):e106230.

13.        Kenyon CR, Tsoumanis A, Schwartz IS. A population's higher-risk sexual behaviour is associated with its average sexual behaviour-An ecological analysis of subpopulations in Ethiopia, Kenya, South Africa, Uganda and the United States. *Epidemics. *2016;15:56-65.
